# A qualitative evaluation of stakeholder perspectives on sustainable financing strategies for ‘priority’ adolescent sexual and reproductive health interventions in Ghana

**DOI:** 10.1186/s12913-024-10743-4

**Published:** 2024-03-26

**Authors:** Evans Otieku, Ama P. Fenny, Daniel M. Achala, John E. Ataguba

**Affiliations:** 1 Department of Public Health, Aarhus University, Aarhus, Denmark; 2 Economics Division, Institute of Statistical, Social and Economic Research (ISSER) , University of Ghana, Accra, Legon Ghana; 3 African Health Economics and Policy Association, Accra, Ghana; 4 Department of Community Health Sciences, Max Rady College of Medicine, Rady Faculty of Health Sciences, University of Manitoba, Manitoba, Winnipeg Canada; 5 Health Economics Unit, School of Public Health and Family Medicine, Health Sciences Faculty,, University of Cape Town, Anzio Road, Observatory, Cape Town, 7925, South Africa; 6 Partnership for Economic Policy, Duduville Campus, Kasarani, Nairobi, Kenya

**Keywords:** Funding strategies, Adolescent, Sexual/reproductive health, Interventions

## Abstract

**Background:**

Adolescent sexual and reproductive health (ASRH) interventions are underfunded in Ghana. We explored stakeholder perspectives on innovative and sustainable financing strategies for priority ASRH interventions in Ghana.

**Methods:**

Using qualitative design, we interviewed 36 key informants to evaluate sustainable financing sources for ASRH interventions in Ghana. Thematic content analysis of primary data was performed. Study reporting followed the consolidated criteria for reporting qualitative research.

**Results:**

Proposed conventional financing strategies included tax-based, need-based, policy-based, and implementation-based approaches. Unconventional financing strategies recommended involved getting religious groups to support ASRH interventions as done to mobilize resources for the Ghana COVID-19 Trust Fund during the global pandemic. Other recommendations included leveraging existing opportunities like fundraising through annual adolescent and youth sporting activities to support ASRH interventions. Nonetheless, some participants believed financial, material, and non-material resources must complement each other to sustain funding for priority ASRH interventions.

**Conclusion:**

There are various sustainable financing strategies to close the funding gap for ASRH interventions in Ghana, but judicious management of financial, material, and non-material resources is needed to sustain priority ASRH interventions in Ghana.

**Supplementary Information:**

The online version contains supplementary material available at 10.1186/s12913-024-10743-4.

## Introduction

Adolescents, a critical mass of the world’s population, have, in the last 29 years since the launch of the International Conference on Population and Development (ICPD), undergone significant demographic, health, and socioeconomic transitions. In 2019, the world had an estimated 1.3 billion adolescents aged 10–19 years, representing a 14.8% increase since 1994, and up to 30% if the age bracket is extended to < 24 years [1]. Compared to other regions, the population of adolescents in sub-Saharan Africa (SSA) has more than doubled over the last three decades and may triple by 2050 [1, 2].

As the population of adolescents increases with growing economies, so are the challenges peculiar to adolescents [3, 4]. Compared to a global average of 5%, approximately 12% of adolescent girls in SSA are forced into early marriages before their fifteenth birthday [5]. Between 1994 and 2017, the prevalence of sexually transmitted infections (STIs), including HIV, gonorrhea, trichomoniasis, and genital herpes among adolescents, increased by 30.3%, equivalent to over 5 million new STIs [6]. Increased adverse events accompanying adolescent sexual and reproductive health (ASRH) like early pregnancy and childbirth are well documented [6, 7]. For example, a multi-country study by the World Health Organization (WHO) shows that about 20% of maternal-related deaths in SSA occur among adolescents [7]. Likewise, in some African countries, more than 40% of adolescents have experienced various forms of intimate partner violence [1]. We argue that the problems adolescents go through are more than we have projected and there are notable disparities across regions and countries in the world.

As of 2021, Ghana had an estimated close to 10 million adolescents aged 10 to 24 years, representing about a 25% increase from 2010 [8]. A joint study by the Ghana Health Service (GHS) and the United Nations Children Fund (UNICEF) indicates that 36% of adolescents in Ghana were sexually active, of which 50% and 13% were either forced or physically coerced, respectively [9]. At the same time, another study shows that unsafe abortion remains the leading cause of maternal deaths for adolescent girls less than 19 years old [10]. In general, published evidence indicates that adolescents are being caught up in essential health service delivery gaps in low-and-middle-income countries (LMIC) as ASRH challenges are overlooked and less prioritized [11, 12]. By WHO estimation, countries in LMIC need approximately US$9.00 per capita to implement one priority ASRH preventive intervention such as contraception, counselling, fertility care, pregnancy-related care, capacity building to prevent intimate partner violence, and unsafe abortion [13]. Deductively, Ghana may require not less than US$10 million for each adolescent to benefit from one priority ASRH preventive intervention annually. The question is how sustainable Ghana can raise such money to continuously provide priority ASRH intervention for the growing adolescent population. Elsewhere, we have addressed the question regarding the funding gap for ASRH interventions [14, 15]. Therefore, this present study by the African Health Economics and Policy Association (AfHEA) is part of an ongoing project on the Economics of Adolescent Sexual and Reproductive Health (EcASaRH) interventions. The aim is to answer the question of how to sustain strategic funding for priority ASRH interventions by drawing on the suggestions and experiences of stakeholder institutions in Ghana. Using the definition by Salam et al. [16], this study defines priority ASRH interventions as effective interventions related to improving ASRH outcomes such as prevention of unintended pregnancies, unsafe abortions, micronutrient supplementation and nutrition for pregnant adolescents. Others may include strategies to prevent substance abuse, early marriages, STIs, and access to needed essential health services, more generally.

## Materials and methods

### Design

This study used a sequential qualitative design [17, 18] involving cross-sectional data collected from key informants to perform thematic content analysis. The design was sequential because we collected data in two phases where Phase 1 data informed how and what data to collect in Phase 2. The study received ethics approval from the Ghana Health Service Ethics Review Committee with reference number GHS-ERC:004/10/2019. Reporting quality and transparency were checked using the consolidated criteria for reporting qualitative research (COREQ) [19].

### Setting

The setting is Ghana, one of two countries in sub-Saharan Africa where the EcASaRH project is being implemented. At the time of this study, the government of Ghana had no specific budget earmarked for implementing priority ASRH interventions. Rather, a composite fiscal allocation is made available to address all health-related problems through the Ministry of Health, the Ghana Health Service, and the National Health Insurance Authority, supported by health aid from bilateral and multilateral development partners. It is public knowledge that fiscal austerity and competing demand for scarce resources, typical in most LMIC settings, mean that priority ASRH interventions are given less attention regardless of the persistent adolescent health problems accompanying the increasing adolescent population.

### Participant and sampling

Participants were purposively invited key informants drawn from multiple institutions in public and private sectors as well as academia/research, civil society organizations, non-governmental organizations, and multi-national development partner institutions. We contacted participants through emails and placed follow-up mobile phone calls on those who did not respond to their emails after 72 h. We obtained their contact information from a list compiled during participant registration to attend an ASRH stakeholder conference organized by the AfHEA in the previous year as part of EcASaRH project activities. In total, 11 participants from 15 invited institutions participated in Phase 1 data collection between December 2022 and January 2023, while additional 25 key informants, making 36 participants in total, partook in Phase 2 data collection on July 4, 2023. Because this study is a qualitative study, the sample size was purposively determined as representative of ARSH stakeholder institutions in Ghana. The invited institutional participation rate was 73% in Phase 1 (11/15) and 100% (19/19) in Phase 2). The 27% non-participation rate in Phase 1 was because four invited participants were unavailable for the interview but participated in Phase 2.

Over 70% of participants served in management capacities as programme managers, monitoring and evaluation officers, budget officers, research fellows, principal nursing officers, operation managers and grant managers. The remaining 30% were in senior management positions as director, founding officer, chief executive, lawmaker/parliamentarian. Additionally, 20 invited adolescents joined the workshop as observers to listen and learn from the expert discussions during the Phase 2 data collection. Table 1 gives an overview of the distribution of invited participants and their institutional affiliations. Regarding gender, Phase 1 had 54.5% (6/11) women participation and Phase 2 had 58.3% (21/36) women participation.

**Table 1:** Distribution of study participants by institutional affiliations

Stakeholders	Phase 1		Phase 2		
	Number of persons invited for KII	Number of persons who participated in KII	Number of invited participants for the workshop	Number of persons who participated in the group discussion	Operational space/sector
Alliance for Reproductive Health Right	2	1	2	2	NGO
DKT International Ghana	2	1	2	2	Private- for-profit
Ghana Health Service	2	1	2	2	Public
Marie Stopes International, Ghana	1	1	3	2	Private
Ministry of Education	4	1	4	1	Public
Ministry of Gender, Children and Social Protection	3	1	3	2	Public
Ministry of Health	3	1	4	3	Public
National Health Insurance Authority	2	0	3	2	Public
National Population Council	1	0	2	2	Public
National Youth Authority	1	1	2	2	Public
Plan International Ghana	1	1	2	1	NGO
Planned Parenthood Association of Ghana	2	1	2	2	NGO
UNFPA	3	1	3	2	DP
UNICEF	1	0	1	1	DP
WHO	2	0	2	1	DP
Academia	-	-	5	4	Public education
Policy Think Tank/Civil Society Organisations	-	-	2	2	CSO
Law maker/Parliamentarian	-	-	1	1	Public
Minister of State	-	-	1	1	Public
Non-participant observers	-	-	20	20*	Students
Total	30	11	64	36	

Note. NGO– non-governmental organisation, DP– development partners, KII– key informant interview, *Participated in the workshop as observers but did not contribute to the group discussions that required expertise and experience relating to financing of ASRH interventions in Ghana

### Data curation and processing

We collected data from key informants through in-person interviews and group discussions. An interview guide developed by the AfHEA in consultation with one senior academic staff of the University of Ghana (see supplementary material) was used to elicit data. Using 10% of the targeted sample, we scheduled four interviews to pilot the instrument for two days to identify potential data incoherence and difficulties in administering the instrument before Phase 1 data collection began. The piloting informed the restructuring of the sub-questions and how to moderate the interview to keep to time, as most participant interviews in Phase 1 took place during working hours. Phase 1 data collection took place face-to-face on an agreed date, time, and location, mainly at participant offices, while four interviews were held using zoom communication service technology. Except for 2 participants who shared office space with other staff, requiring that the interview be held in the presence of their colleague and at their convenience, we held the remaining interviews in Phase 1 privately. The Phase 1 data collection gathered data on funding gaps for ASRH interventions in Ghana. Participants were also asked to suggest ways to address the funding gap. However, not enough data points were generated from Phase 1 on ways to address the funding gaps. Consequently, AfHEA invited all key informants from ASRH stakeholder institutions to an organized information workshop at the University of Ghana Medical Centre on July 4, 2023, to share knowledge and discuss sustainable ways to finance priority ASRH interventions in Ghana, which form Phase 2 of the data collection. For each participant interviewed, we determined data saturation if responses to specific questions were reoccurring, and we were convinced that we had obtained enough data points to justify our study conclusion. As participants were adults, educated and employed in various senior management positions at their designated institutions, we did not collect further background data on these variables, except noting their gender for equal representation.

We sought permission from participants to collect data using digital audio recording devices (smart mobile phone recorders for Phase 1 and laptop computer for Phase 2) supplemented by interviewer notes on relevant points. Phase 1 interviews lasted 20 min on average, while Phase 2 lasted about 2 h as each group participant was allowed to speak followed by additional 10-minute coffee break after every hour group discussion. Phase 1 data collection was collected by an independent Ghanaian consultant, while in Phase 2, a trained staff of the AfHEA assisted the consultant, given that we grouped the workshop participants into two for the discussion and data collection. Both data collectors had training and experience managing other funded qualitative data collection in Ghana and other African countries. The consultant managed data coding alone and stored the audio recording for further reference.

We established data reliability in two ways. First, at the end of each session of the interview in Phase 1, we made sure participants validated what data we collected by providing a verbal summary of the notes and major points taken. Second, all Phase 1 participants attended the workshop in Phase 2, and we briefed them on the results of Phase 1 through an information session that allowed feedback and validation. Again, at the end of the workshop, we assembled all participants in one conference room and summarized the main points taken from the breakout group discussion.

### Data analysis

Thematic content analysis of primary data was performed. Data points were assigned unique color codes and reclassified into 4 major and 2 minor themes using the number of participants as a weight to determine the order of significance of each theme as embedded in grounded theory of qualitative science [18]. Two of the investigators established the qualitative themes from the audio-recorded interview and field notes taken. The process involved listening to each recorded audio file twice to extract the themes in participant responses to each question and grouping similar themes from different participants. We transcribed the audio file using Microsoft Word and tabulated the themes in Microsoft Excel. Having ensured participants validated the data for reliability, we subject the transcript and result to internal quality control checks through double peer review of the themes and quotations extracted from the audio file to buttress each point in the result. Final draft manuscript was reviewed and approved by all the investigators. As the interviews and discussions with study participants during data collection were performed in English Language, no technical language interpretation of data was required before the analysis.

## Results

We asked participants to recommend and give reasons for at least one strategy they believed could help the government to sustainably finance ASRH interventions in Ghana. Overall, participant responses to the question were grouped into four major themes in descending order of significance using the number of observations as measured weight (Table 2).

**Table 2:** Stakeholder perspectives on strategies to sustain financing for priority ASRH interventions

Strategies	Number of participants
Tax-based financing	12
Policy-based	9
Need-based	8
Implementation-based	5
Mobilizing support from religious bodies	1
Mobilizing funds from youth sporting activities	1
Total	36

### Tax-based strategies

Taxed-based strategies emerged the most dominant theme for sustainable financing of priority ASRH interventions in Ghana. Twelve participants shared this view and suggested ways the government could mobilize sustainable domestic revenue for ASRH interventions. One of the 12 participants mentioned that removing taxes on sanitary pads alone could keep approximately 3.8 million adolescent girls in school annually and avert tendencies where needy young girls do not go to school because they cannot afford sanitary wares during their menstrual cycle. She added that:

Tax-based strategies for sustainable financing of priority ASRH interventions may involve widening the tax net for more revenue, recalibrating existing taxes, or removing some retrogressive taxes. In Ghana, nine out of ten adolescent girls regularly stay out of school between 2 and 5 days every month because they cannot afford sanitary pads during their menstrual cycle. Simply removing taxes on sanitary pads will make them affordable, keep our daughters in school, and reduce the likelihood of them engaging in risky sexual behaviours because they are home. Though such a strategy will marginally reduce tax revenue, the macroeconomic benefit will be enormous as the demand and supply-side effects will be positive for producers and consumers, including adolescent girls (Key informant, NGO, July 4, 2023).

Two other participants recommended government recalibrate some taxes to provide sustainable funding for priority ASRH interventions. They agreed that government could mobilize significant sustainable domestic revenue for the Ghana Health Service to implement priority ASRH interventions by allocating 1% out of the 5% taxes on Minerals and Mining Operations in Ghana. One participant reiterated that:

Natural mineral resources are blessings from God and are meant for all to benefit, including generations unborn. As adolescents constitute approximately a third of the country’s population and the future of the country, it will be right that a percentage of revenue from mineral royalties go to fund adolescent health interventions to avert unintended consequences associated with ASRH (Key informant, Development Partner, July 4, 2023).

Another tax-based strategy mentioned was for the government to redirect the 1% COVID-19 levy on imported goods and services to finance ASRH interventions. This financing strategy, according to participants, was against the backdrop that the World Health Organization no longer consider COVID-19 a global/public health threat, which renders the COVID-19 Health Recovery Levy, 2021 (Act 1068) unconstitutional for raising revenue to support COVID-19-related expenditures. Most participants agreed with this strategy. A key informant had this to say:

It will not be out of place to redirect the COVID-19 levy on imported goods and services to address the health needs of adolescents. As a country, we should evaluate our priorities and consider health financing, such as those for ASRH, an economic investment to drive macroeconomic prosperity for Ghana in the long term. (Key informant, NGO, July 4, 2023).

Four key informants agreed to a proposition that if the government had challenges with limited domestic revenue for financing priority ASRH service, it could adapt appropriate technology to broaden the tax net to collect more taxes from the overwhelming informal economy where people make profits and incomes without paying taxes to the government. Excerpts from two participants are presented below.

Indeed, the government lack strategy and commitment to mobilize tax revenue from the informal sector. If the government had mobilized tax revenue from the informal sector, we may not be here deliberating on ways to address funding gaps for priority ASRH interventions. One difference between Ghana and advanced countries that are meeting the health needs of adolescents is that health interventions by the state are funded from tax revenue collected from both formal and informal sectors (Key informant, Ministry of Education, January 5, 2023).

In some parts of the world, tax revenue is a sustainable source of domestic revenue to finance public health interventions like ASRH. In Ghana, it appears the government uses more than 60% of tax revenue to pay salaries and allowances of public sector workers. Meanwhile, our needs as a country outweigh the revenue we mobilize domestically. Therefore, drawing in taxes from the informal sector may be one solution to raise domestic revenue for ASRH interventions without always relying on health aid and grants from our development partners (Key informant, Academia/Research, July 4, 2023).

### Policy-based strategies

Participants recommended three policy-based financing strategies for ASRH interventions. First, one participant said having a long-term national policy and legal framework that entrenches sustainable fiscal allocation to finance specific ASRH intervention programmes is a prerequisite. The participant reiterated that:

There is a need for policymakers to have legal provisions and policies that stipulate strategic funding sources and consistent annual allocations for the Ghana Health Service and other service providers to implement ASRH interventions. Civil society organizations representing the interest of adolescents in Ghana could spearhead advocacy or national debate for this effort to allow parliament to make a law to that effect (Key informant, Development Partner, July 4, 2023).

Second, another participant added that because female adolescents are more vulnerable, policies that promote gender-sensitive fiscal allocation for ASRH interventions could be one solution if Ghana has limited resources. The participant said that:

It will be good that government prioritize policy-based interventions that insulate vulnerable adolescent groups when allocating scarce resources for ASRH interventions. Such policy considerations may include a national establishment of community gender-based clubs and rehabilitation centers where girls could meet professional peer counsellors to engage in informal conversations regarding ASRH. This intervention could be a modest cost-saving way of insulating young girls from stigma, suicidal thoughts and averting many other problems adolescents encounter (Key informant, Public Sector, January 11, 2023).

Third, a different participant proposed a deliberate involvement of the private sector to support government efforts in financing ASRH interventions. Five participants agreed that the government cannot fund every intervention. Therefore, policies promoting public-private partnerships for ASRH may be a suitable alternative to sustain funding for priority ASRH interventions. The participant said that:

Some medium and large-scale enterprises may be willing to support ASRH interventions through corporate social responsibility because adolescents are their target market. However, the government must identify such organizations and enter into a long-term agreement to support adolescents with critical health needs. Often this support may not be financial but adequate supplies such as sanitary pads and nutrition supplements to mitigate adolescent health challenges (Key informant, Private Sector, Ghana, July 4, 2023).

### Need-based strategies

Need-based strategies for sustainable financing of ASRH interventions were the third dominant theme recommended by stakeholders. Six participants agreed that adolescents have several needs to improve their health and well-being, but some may be to avert adverse outcomes and requires national attention. The following were some suggestions by two participants to improve funding of priority ASRH interventions in Ghana.

Need-based strategies have proven to be a reliable strategy in resource-constraint settings. For instance, the government must financially equip ASRH service providers to provide targeted need-based services for adolescents who cannot afford life-saving health services when needed. It is one plausible way to cut costs by focusing interventions on adolescents who cannot afford essential services (Key informant, Public Sector, July 4, 2023).

There is so much wastage and corruption in public sector financing. Sustained reliable financing of ASRH interventions is possible if the government can reduce wastage by cutting down unnecessary spending and corruption in the public sector (Key informant, NGO, July 4, 2023).

### Implementation-based strategies

Five participants suggested implementation-based strategies to improve sustainable financing for priority ASRH interventions. The strategies included budget and expenditure tracking of funded ASRH interventions through effective monitoring and evaluation. Others were public and private partnerships, using appropriate technology to deliver preventive intervention at reduced cost and building a culture of credibility and transparency when implementing ASRH interventions to attract financial support from development partners. For example, one participant representing a development partner institution shared the following:

Countries worldwide are developing budget, expenditure and programme tracking systems to reduce duplication and save limited funds for cost-effective interventions. For example, United Nation Agencies have systems to track resource use and programme implementations to reduce costs. The government of Ghana can do the same through the Ministry of Monitoring and Evaluation if they have not started already (Key informant, Development Partner, July 4, 2023).

Reiterating a similar point, a second participant from a non-governmental organization said:

Building a culture of credibility and transparency can attract sustainable funding for ASRH interventions. For us in the NGO sector, that has been a crucial factor in attracting competitive grants and health aid from Foundations and multinational financial institutions. State-implementing institutions and service providers can do the same for ASRH (Key informant, NGO, July 4, 2023).

A third participant suggested the need for government and ASRH service providers to generate reliable data through actuarial studies that quantify the potential cost-benefit of ASRH interventions. The participant believed lobbying politicians and policymakers for funding requires accurate data. Three other participants who shared the same view said:

Implementation science data can help reduce ASRH-associated costs by discontinuing interventions that are not cost-effective and prioritizing those that are cost-effective (Key informant, Development Partner, July 4, 2023).

Interventions like those to prevent unsafe abortions and forced adolescent early marriages should be co-created with communities to encourage continued communal support/ownership at relatively reduced costs during and beyond the intervention lifecycle (Key informant, NGO, January 11, 2023).

Cutting costs is another way to sustain funding for ASRH interventions. Appropriate digital technologies could be a cheaper alternative to delivering preventive ASRH services. However, the appropriateness of such technologies should be piloted locally before a national rollout. Telecommunication companies can facilitate this process as part of corporate social responsibility to reduce the financial burden from the state (Key informant, Public Sector, January 2, 2023).

### Support of religious groups

Besides the strategies mentioned above, two other participants said Ghana was underestimating the potential support of religious groups in terms of their ability to offer counselling and material support for ASRH interventions. One of the participants shared the following:

Issues of child marriages, risky sexual behaviours among adolescents, unintended pregnancy and unsafe abortions are issues of morality that religious groups could help resolve through adolescent counselling sessions, biblical teachings, and material support like donation of sanitary wares adolescents in remote villages. It will be less costly if the government appeal to the Christian Council of Ghana to discuss ways in which, for example, churches could contribute to reducing adverse outcomes associated with ASRH at relatively little or no cost to the government (Key informant, Private Sector, January 11, 2023).

Another participant gave an example of the generous contributions of religious groups during COVID-19. She said:

Government and institutional stakeholders should appeal to religious groups to offer support like they did to support the national COVID-19 Trust Fund. One thing is that some ASRH interventions may require material and non-material resources that religious groups can support by appealing to their congregants working in industries and other businesses (Key informant, NGO, July 4, 2023).

### Funding through adolescent sporting activities

One participant suggested adolescents themselves can facilitate fundraising efforts through annual sporting activities coordinated by the Ministry of Youth and Sports.

Organized adolescent sporting activities can generate revenue annually to support ASRH interventions for vulnerable adolescents. The Ministry of Youth and Sports can coordinate this effort to support priority ASRH interventions. Alternatively, a percentage of every income from general sporting activities could be dedicated to financing priority ASRH interventions as adolescents dominate the sporting sector (Key informant, Policy Think Tank, July 4, 2023).

### Summary of findings

Table 3 presents a snapshot of the major findings/themes from the study. It indicates that both financial and material resources are needed to complement each order to close the funding gap for priority ASRH interventions in Ghana.

**Table 3:** Summary of perceived sustainable financing strategies for priority ASRH interventions in Ghana

Category of strategy	Recommended sustainable ASRH financing approaches	Participant institutional representation
Tax-based strategies	The government should widen tax net to mobilise support from the informal sector, re-align/recalibrate existing tax handles in favour of ASRH interventions, and remove taxes on sanitary pads for adolescent girls.	A non-governmental organization (NGO), Development Partner (DP), Public sector, and academia/research
Policy-based strategies	Parliamentarians and Civil Society Organizations should work to promote entrench legal provisions and policies that guarantee long-term domestic financing for priority ASRH interventions and adopt policies that embrace multiple private sector partnership support for ASRH interventions.	The private sector, public sector, DP
Need-based strategies	The government and other institutions providing ASRH services should deploy needs assessment mechanisms when allocating scarce resources and services for ASRH interventions. They should allow those who can afford to pay for the service pay for them and those who cannot afford to be given free or subsidised services.	Hospital-based service providers, NGOs, public sector
Implementation-based strategies	Some interventions must be co-created by ASRH service providers and target beneficiaries, and data driven to reduce costs and garner support of policymakers for domestic resource allocations for ASRH interventions. Deliver preventive interventions via digital platforms for LMICs.	NGO, DP, Private sector
Support of religious groups	As evidenced by religious group support for COVID-19 interventions in Ghana, the government can leverage the Christian Council of Ghana to support priority ASRH interventions.	Private sector, NGO
Funding through adolescent sporting activities	The Ministry of Youth and Sports should organize national adolescent sporting activities to generate sustainable revenue for priority ASRH interventions.	Civil society organization

## Discussion

As countries in sub-Saharan Africa face an imminent escalation of the adolescent population amidst scarcity of resources to address the funding gap for ASRH interventions due to donor fatigue [20–22], this qualitative study explored feasible resource mobilization strategies to sustain funding for priority ASRH interventions. Several financing strategies were recommended by key informants purposively selected due to their role in offering direct and indirect ASRH services in Ghana. In reverse order of significance, dominant financing strategies included tax-based, policy-based, need-based, and implementation-based approaches. Others were state mobilization of support from religious groups and revenue from mainstream adolescent sporting activities.

The suggestion by participants that tax-based financing approaches could be a feasible option for financing ASRH interventions in Ghana was congruent with a study by the World Health Organization [13]. In the WHO study, tax-based financing was mentioned as the most reliable domestic resource mobilization strategy compared to need-based approaches because it involves relatively less administrative cost and is easy to mobilize. State revenue collection institutions can mobilize, allocate and account for tax revenue given to ASRH service providers and at the same time benefits can reach every target group. For example, approximately 6% of the estimated US$190.27 million worth of royalty from crude oil in Ghana for 2023 [23] could fund one priority ASRH intervention programme each year, as indicated by WHO [13].

As one participant pointed out, a lack of strategy and commitment by government to mobilize tax revenue from informal sector contributes to the lack of funding for priority interventions like those for ASRH. We argue from societal perspective that a major reason this challenge persist is the over politicization of taxation and the persistent need to please citizens for fear of government losing political power to opposition political party if they commit to implementing such taxes. Unlike the formal sector, there is limited data on the informal economy for tax purposes compounded by irregular earnings in the informal sector and the fear that disclosing income to tax authorities will affect their economic fortunes. Nevertheless, the digitalization policy of government and the introduction of 1% digital tax on financial transaction is one way government can mobilize tax revenue from the informal sector. Perhaps, it is a question of whether government can properly identify how much of the digital taxes come from the informal economy or informal sector employees. Moreover, the decision by the government to waive taxes on locally produced sanitary wares and import duties on raw materials for the same as reported in the 2024 Budget Statement and Economic Policy by the Ministry of Finance is commendable [24]. The tax waiver means less production costs and millions of adolescent girls can afford sanitary wares during their monthly cycle.

We argue that when taxpayers are overburdened with taxes and resisting paying more for priority interventions, need-based financing may be an alternative strategy to reduce costs through exemption policies [25, 26]. Thus, most adolescents who can afford essential ASRH services should pay to sustain free provision for those who cannot afford them. Regardless of the strength associated with need-based financing, its application could be cumbersome due to bias in allocation criteria [27].

Some of the proposed implementation-based financing strategies in this present study build on the principal-agent theory of performance-based financing, where scarce resource allocations favor cost-effective intervention programmes [28]. Investors, development partners, and policymakers acting as principal financiers may be willing to provide financial support for priority ASRH service providers (agents) if they have reasons to believe there will be prudent use for their investments. Irrespective of how simple this financing strategy may seem, there could be setbacks in implementing new interventions that are yet to generate data to show evidence of effectiveness, requiring that other sustainable financing strategies should run parallel to performance-based financing [29]. Therefore, implementation-based financing strategies may not be the most suitable when implementing new priority ASRH interventions [30, 31].

Besides the recommended conventional sources of sustainable financing for priority ASRH interventions, participants believed unconventional sources like support from religious groups could complement existing financing sources. The belief was that religious groups made generous cash and kind donations to support the national COVID-19 Trust Fund during the global pandemic. Therefore, governments and other stakeholder institutions could solicit similar support to fund ASRH interventions. Whether this financing source is feasible may depend on factors like accountability, which became a topical public discussion because of the lack of transparency regarding COVID-19 expenditures in Ghana and elsewhere [32–34].

As another participant indicated, the government can generate revenue through adolescent sporting activities like annual fun games, in which tickets sold can generate revenue for ASRH intervention. If well-coordinated by the Ministry of Youth and Sports, such initiatives may attract sponsorship from corporate Ghana to support the most vulnerable adolescents. In summary, sustainable resource mobilization for priority ASRH interventions may come in several forms, but prudent management of such resources is crucial to achieving the intended purpose.

The strength of this study hinges on the quality and reliable data triangulated from a cross-section of participants with working knowledge and experience regarding funding for adolescent sexual and reproductive health interventions. To the best of our knowledge, the study contributes to the literature by identifying, evaluating, and documenting scientific evidence on ways to sustain funding for ASRH services to improve the well-being of vulnerable adolescents in an LMIC setting. Regarding limitations, this study adopts a qualitative design using a limited sample of participants, which limits the generalizability of the findings.

### Electronic supplementary material

Below is the link to the electronic supplementary material.

Supplementary Material 1

Supplementary Material 2

## Data Availability

Data used for this research are available upon request to the corresponding author.

## Declarations

### Reflexivity statement

This study was conducted by four investigators comprising three males and one female with institutional affiliations in Ghana, Kenya, South Africa, Denmark and Canada. APF and JEA holds PhD in Health Economics and occupy senior academic positions, EO is an early career researcher and holds PhD in Health Economics, and DMA is an administrative staff of the AfHEA. EO conducted the interviews with the support of DMA. Before the data collection, we informed participants about the team of investigators and the study objective, and knew the mandate of participant institutions as ASRH stakeholders.

## References

[1] Liang M, Simelane S, Fortuny Fillo G, Chalasani S, Weny K, Salazar Canelos P, Jenkins L, Moller AB, Chandra-Mouli V, Say L, Michielsen K, Engel DMC, Snow R (2019). The state of adolescent sexual and Reproductive Health. J Adolesc Health.

[2] United Nations, Department of Economic and Social Affairs PD. World population prospects 2019. Available at: https://population.un.org/wpp/, Accessed July 21, 2023.

[3] Melesse DY, Mutua MK, Choudhury A, Wado YD, Faye CM, Neal S, Boerma T (2020). Adolescent sexual and reproductive health in sub-saharan Africa: who is left behind?. BMJ Glob Health.

[4] Chandra-Mouli V, Armstrong A, Amin A, Ferguson J (2015). A pressing need to respond to the needs and sexual and reproductive health problems of adolescent girls living with HIV in low‐and middle‐income countries. J Int AIDS Soc.

[5] United Nations. Special edition to the sustainable development goals progress report. Report of the Secretary-General, Supplementary Information. New York. 2019.

[6] Institute for Health Metrics and Evaluation (IHME). GBD database. Available at: http://ghdx.healthdata.org/gbd-results-tool, Accessed July 20, 2023.

[7] Ganchimeg T, Ota E, Morisaki N (2014). Pregnancy and childbirth outcomes among adolescent mothers: a World Health Organization multicounty study. BJOG.

[8] Ghana Statistical Service. (2021). Ghana 2021 population and housing census report: General report volume 3B. https://statsghana.gov.gh/gssmain/fileUpload/pressrelease/2021%20PHC%20General%20Report%20Vol%203B_Age%20and%20Sex%20Profile_181121.pdf.

[9] Ghana Broadcasting Corporation. 36% of adolescents in Ghana are sexually active, study reveals. GBC, Accra. 2023. Available at: https://www.gbcghanaonline.com/general/36-of-adolescents/2023/, Accessed July 19, 2023.

[10] World Health Organization. Universal health coverage for sexual and reproductive health in Ghana, WHO, Geneva, Switzerland. 2021. Available at: https://apps.who.int/iris/bitstream/handle/10665/350888/WHO-SRH-21.17-eng.pdf, Accessed July 25, 2023.

[11] Engel DMC, Paul M, Chalasani S, Gonsalves L, Ross DA, Chandra-Mouli V, Cole CB, de Carvalho Eriksson C, Hayes B, Philipose A, Beadle S, Ferguson BJ (2019). A Package of sexual and Reproductive Health and rights interventions-what does it Mean for adolescents?. J Adolesc Health.

[12] Ravindran TKS, Govender V (2020). Sexual and reproductive health services in universal health coverage: a review of recent evidence from low- and middle-income countries. Sex Reprod Health Matters.

[13] World Health Organization. Universal health coverage for sexual and reproductive health, Geneva WHO, Switzerland. 2020. Available at: https://apps.who.int/iris/bitstream/handle/10665/331113/WHO-SRH-20.1-eng.pdf, Accessed July 25, 2023.

[14] Otieku E, Fenny AP, Achala DM, Ataguba JE, Obse AG. 2023. Funding strategies, gaps, and sustainable resource mobilization for adolescent sexual and reproductive health interventions in Ghana. *Evidence Brief*. August 2023. Available at: https://afhea.org/en/ecasarh-project-policy-briefs/.

[15] Otieku E, Fenny AP, Achala DM, Ataguba JE, Obse AG. 2023. Understanding the implication cost of priority adolescent sexual and reproductive health interventions in Ghana. *Evidence Brief*, August 2023. Available at: https://afhea.org/en/ecasarh-project-policy-briefs/.

[16] Salam RA, Das JK, Lassi ZS, Bhutta ZA (2016). Adolescent Health interventions: conclusions, evidence gaps, and Research priorities. J Adolesc Health.

[17] Morse JM (2010). Simultaneous and sequential qualitative mixed method designs. Qualitative Inq.

[18] Creswell JW (2014). Research design: qualitative, quantitative, and mixed methods approaches.

[19] Tong A, Sainsbury P, Craig J (2007). Consolidated criteria for reporting qualitative research (COREQ): a 32-item checklist for interviews and focus groups. Int J Qual Health Care.

[20] Hamilton J, Ivker R (1997). Donor fatigue hits family planning in developing world. Lancet.

[21] Awadari AC (2020). Donor fatigue phenomenon: Trend and circumvention in Northern Ghana. Int J Financial Acc Manage.

[22] Gilbert K, Tenni B, Lê G (2019). Sustainable transition from donor grant financing: what could it look like?. Asia Pac J Public Health.

[23] Republic of Ghana. Mid-year fiscal policy review of the 2023 budget statement and economic policy of the government of Ghana. July 31, 2023. https://mofep.gov.gh/sites/default/files/budget-statements/2023-Mid-Year-Policy-Review_1.pdf.

[24] Republic of Ghana. The budget statement and economic policy of the government of Ghana for the 2024 financial year, November 2023, https://mofep.gov.gh/publications/budget-statements.

[25] Kephart G, Asada Y (2009). Need-based resource allocation: different need indicators, different results?. BMC Health Serv Res.

[26] Kaufman BG, Jones KA, Greiner MA, Giri A, Stewart L, He A, Clark AG, Taylor DH, Bundorf MK, Whitaker RG, Van Houtven CH, Higgins A (2023). Health Care use and spending among need-based subgroups of Medicare beneficiaries with full Medicaid benefits. JAMA Health Forum.

[27] Radinmanesh M, Ebadifard Azar F, Hashjin aghaei (2021). A review of appropriate indicators for need-based financial resource allocation in health systems. BMC Health Serv Res.

[28] Paul E, Bodson O, Ridde V. What theories underpin performance-based financing? A scoping review. *J Health Organ Manag*. 2021;ahead-of-print(ahead-of-print). 10.1108/JHOM-04-2020-0161. 10.1108/JHOM-04-2020-0161 33463972

[29] Waithaka D, Cashin C, Barasa E (2022). Is performance-based financing A pathway to Strategic Purchasing in Sub-saharan Africa? A synthesis of the evidence. Health Syst Reform.

[30] Witter S, Bertone MP, Diaconu K, Bornemisza O (2021). Performance-based Financing versus unconditional direct facility financing - false dichotomy?. Health Syst Reform.

[31] Fretheim A, Witter S, Lindahl AK, Olsen IT (2012). Performance-based financing in low- and middle-income countries: still more questions than answers. Bull World Health Organ.

[32] Ghana Web. Auditing of pandemic expenditure needed for transparency and accountability. https://www.ghanaweb.com/GhanaHomePage/NewsArchive/Auditing-of-pandemic-expenditure-needed-for-transparency-and-accountability-1420021, Accessed August 6, 2023.

[33] COVID Transparency and Accountability Project. Ghana’s COVID-19 Fund Management lack transparency. https://myjoyonline.com/ghanas-covid-19-funds-management-lack-transparency-ctap/, Accessed August 6, 2023.

[34] Schaaf M, Boydell V, Van Belle S, Brinkerhoff DW, George A (2020). Accountability for SRHR in the context of the COVID-19 pandemic. Sex Reprod Health Matters.

