# Supplementary material for: A qualitative evaluation of stakeholder perspectives on sustainable financing strategies for ‘priority’ adolescent sexual and reproductive health interventions in Ghana
Source: BMC Health Serv Res. 2024 Mar 26;24:373. doi: 10.1186/s12913-024-10743-4 (PMC10964666; doi:10.1186/s12913-024-10743-4)
Supplement: Supplementary file 1 — Supplementary Material 1 [file 12913_2024_10743_MOESM1_ESM.docx]

# Supplementary Material 1: Interview Guide

**STUDY TOOL: Key informant interviews**

**Assessment title - Economics of Adolescent Sexual and Reproductive Health (ECASARH) Interventions in Ghana**

**Background**

As key partner institutions involved in the implementation of adolescent sexual and reproductive health interventions in Ghana, we are delighted for your continued participation in the ECASARH project by the African Health Economics and Policy Association. The next phase of ECASARH project is to assess resource needs, funding gaps, and identify sustainable financing strategies to implement priority ASRH interventions in Ghana using a multi-component, multisectoral approach. Following the stakeholders' meeting that took place at the Mensvik Hotel in Accra on 7th September 2022, it was largely agreed funding gaps were stalling ASRH interventions. This was due to donor-driven objectives of ASRH and low funding from governments generally linked to sustainable development. Also, the cost estimates from the priority ASRH interventions in Ghana suggest instability in ASRH funding given the erratic fluctuations in funding of interventions over the years.

All stakeholders agreed that changes in international priorities could have dire consequences on funding of ASRH interventions, which could derail the gains made in providing ARSH services in Ghana. This questionnaire is to gather information on the funding gaps and financing strategies from key stakeholders supporting the funding and implementation of ASRH services in the country.

1. **Overview of the stakeholder's area of operation**

- What is the stakeholder’s (participant institution) area of operation and role in ASRH?
- Can you provide any specific example of responsibility/interest to indicate stakeholders’ role in ASRH?
- Does the stakeholder/participant engage other agencies involved in ASRH services?

1. **Funding gap for ASRH services**

- Could you share your opinion on trend in ASRH funding gap for the last 5 years - % per year?
- What are the main sources of funding ASRH services in Ghana?

1. **Key challenges/reasons for the funding gap**

- Any systemic challenges
- Any operational challenges
- Other challenges (provide specific examples)

1. **Sustainable Financing strategy for ASRH interventions in Ghana**

- Any national/local financial strategy – existing or planned (ask for policy documents) and who is/was involved in the design of the strategy?
- What are the Challenges with the implementation of ASRH financing strategy?
- Can you please mention and explain at least one innovative/sustainable financing strategies you believe can be implemented to improve funding for ASRH interventions in Ghana?
